# Supplementary material for: PIF7 is a master regulator of thermomorphogenesis in shade
Source: Nat Commun. 2022 Aug 29;13:4942. doi: 10.1038/s41467-022-32585-6 (PMC9424238; doi:10.1038/s41467-022-32585-6)
Supplement: Supplementary file 1 — Supplementary Information [file 41467_2022_32585_MOESM1_ESM.pdf]

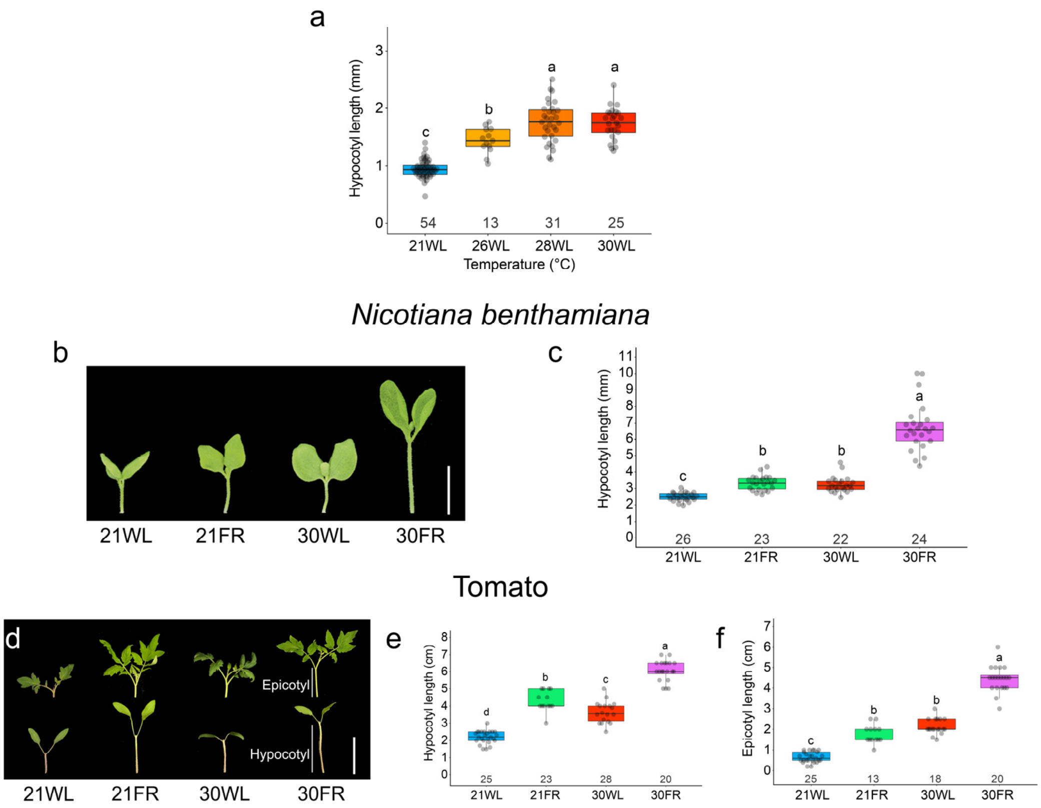

Supplementary Figure 1: The synergistic growth response to low R/FR and warm temperature in tomato and *Nicotiana Benthamiana*.

- **a.** Hypocotyl length of 6-day-old Arabidopsis wild-type seedlings grown for three days at 21°C continuous white light (70 µmol m^{-2} s^{-1}) and moved to the indicated temperature in the same light condition. The number of seedlings (n) is shown under each box.
- **b.** Representative images of 10-day-old *Nicotiana benthamiana* seedlings, grown at 21°C for six days, and then moved to the indicated conditions. Light: constant white light (70 µmol m^{-2} s^{-1}) with or without supplemented Far-Red (FR- R/FR = 0.6). Scale bar = 5 mm.
- **c.** Hypocotyl length measurements of *Nicotiana benthamiana* seedlings shown in **b**. The number of seedlings (n) is shown under each box.
- **d.** Representative images of 21-day-old tomato seedlings grown at 21°C for 9 days and then moved to the indicated conditions. Light: long day white light (16h light:8h dark , white LED, 100 µmol m^{-2} s^{-1}) with or without supplemented Far Red; (R/FR = 0.6). Scale bar = 2 cm.
- **e.** Hypocotyl length measurements of tomato seedlings are shown in **d**. The number of seedlings (n) is shown under each box.
- **f.** Epicotyl length (the segment between the cotyledons and the first leaf) measurements of tomato seedlings shown in **d**. The number of seedlings (n) is shown under each box.

In **a**, **c**, **e,** and **f,** different letters denote statistical differences ($p < 0.05$) among samples as assessed by one-way ANOVA and Tukey HSD test. Boxes indicate the first and third quartiles and the whiskers indicate the minimum and maximum values, the black lines within the boxes indicate the median values and gray dots mark the individual measurements.

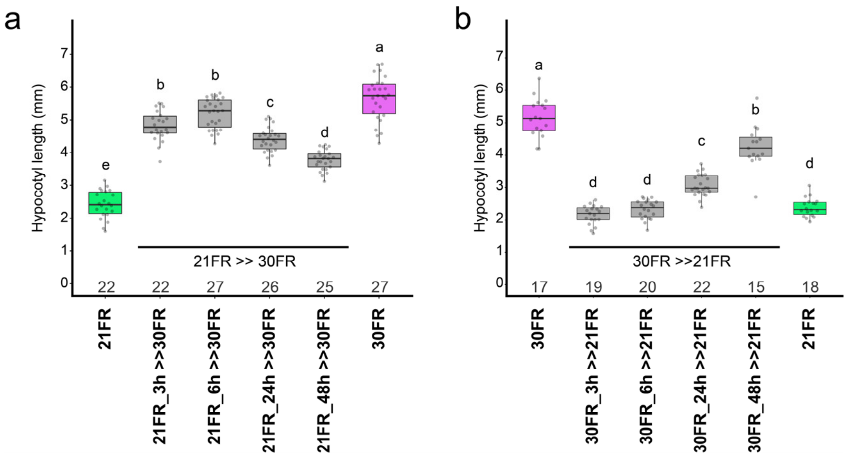

Supplementary Figure 2: The hypocotyl elongation growth response to 30FR or to 21FR is temperature-labile in the first 48h of treatment.

a, b. Hypocotyl length of 6-day-old Arabidopsis wild-type seedlings grown for three days at 21°C constant white light (70 μmol m^{-2} s^{-1}), then moved to the indicated conditions. In gray, the seedlings were transferred from 21FR to 30FR (**a**) or from 30FR to 21FR (**b**), after the indicated time and stayed there for the rest of the experiment. Different letters denote statistical differences ($p < 0.05$) among samples as assessed by one-way ANOVA and Tukey HSD test. (**a,b)** The number of seedlings (n) is shown under each box. Boxes indicate the first and third quartiles and the whiskers indicate the minimum and maximum values, the black lines within the boxes indicate the median values and gray dots mark the individual measurements.

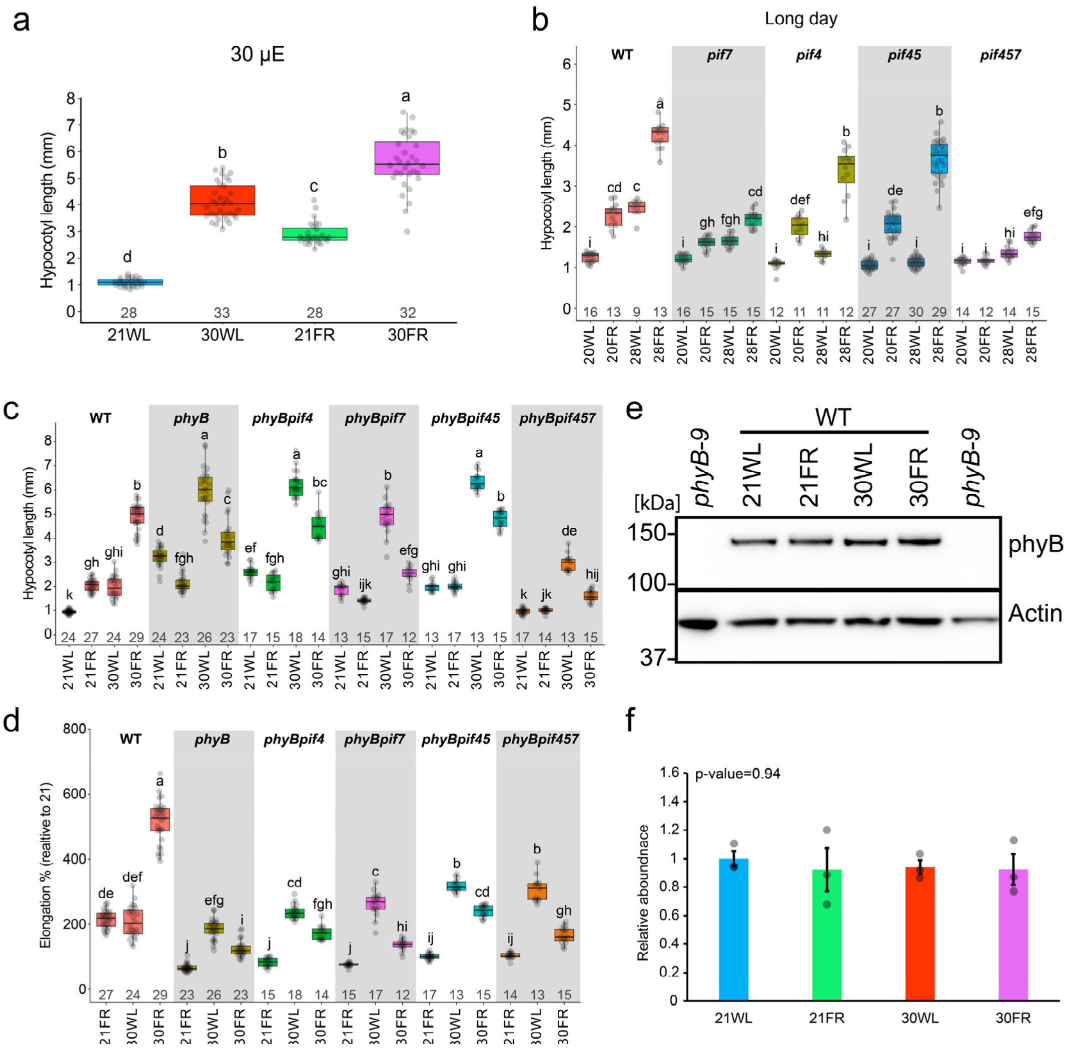

Supplementary Figure 3: The role of PIFs in the synergistic response of low R/FR and warm temperature in long-day conditions and their interaction with *phyB* mutant.

- **a.** Hypocotyl length of 6-day-old Arabidopsis wild-type seedlings grown in 21°C with constant low simulated white light (WL- 30 µmol m^{-2} s^{-1}) for three days and then moved to 30°C (30WL, 30 µmol m^{-2} s^{-1}), 21°C with low R/FR (21FR, R/FR = 0.6, PAR=30 µmol m^{-2} s^{-1}), 30°C with low R/FR (30FR, R/FR = 0.6, PAR=30 µmol m^{-2} s^{-1}) or kept at 21°C (21WL, PAR=30 µmol m^{-2} s^{-1}) for three more days. The number of seedlings (n) is shown under each box.
- **b.** Hypocotyl length of 6-day-old Arabidopsis seedlings of the indicated genotypes grown for three days at 20°C, then moved to the indicated conditions. Light: long-day white light (16h light:8h dark, fluorescent bulbs, 70 µmol m^{-2} s^{-1}) with or without low R/FR (0.6); Temperature- constant 20°C or 28°C. The number of seedlings (n) is shown under each box.
- **c.** Hypocotyl length of 6-day-old Arabidopsis *phyB pif* mutant seedlings. The number of seedlings (n) is shown under each box. The growth condition is the same as described in Figure 1a, PAR= 70 µmol m^{-2} s^{-1}.
- **d.** Elongation percentage based on the hypocotyl length shown in **c**. The elongation percentage was calculated by dividing the hypocotyl length of each seedling by the average length at 21WL. The number of seedlings (n) is shown under each box.
- **e.** Representative immunoblot showing the abundance of phyB protein levels using anti-phyB antibody. Total protein extract collected from 3-day-old WT Arabidopsis seedlings grown in 21WL

(70µmol m^{-2} s^{-1}) plus three hours in the stated conditions. Anti-ACTIN blots are shown as loading controls. We repeated the experiment three times with similar results.

f. Quantification of phyB protein normalized to ACTIN protein levels and to 21WL. Shown are the average values, from three independent experiments, +/- SE and the p-value of one-way ANOVA test.

In **a-d,f**, different letters denote statistical differences ($p < 0.05$) among samples as assessed by one-way ANOVA and Tukey HSD test. Boxes indicate the first and third quartiles and the whiskers indicate the minimum and maximum values, the black lines within the boxes indicate the median values and gray dots mark the individual measurements.

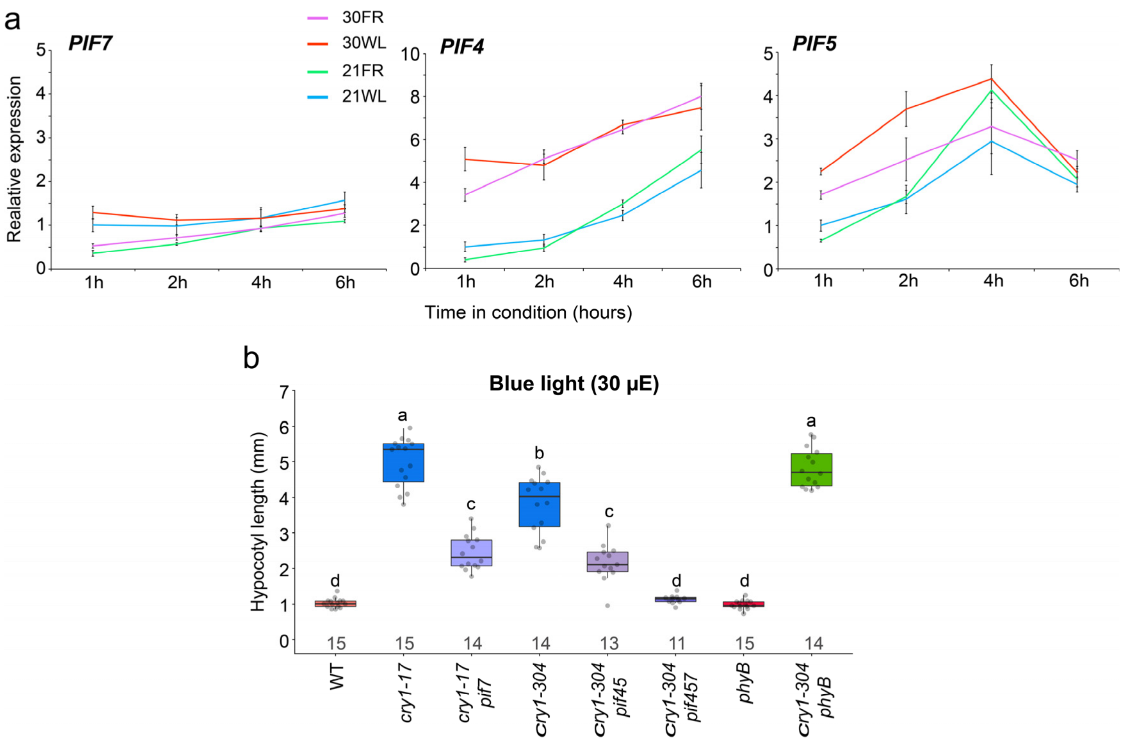

Supplementary Figure 4: *PIF* expression in response to 21FR, 30WL, or 30FR and the role of *PIF7* downstream of *CRY1* in monochromatic blue light.

- **a.** Expression of *PIF4*, *PIF5*, and *PIF7* in 3-d-old Arabidopsis seedlings grown in constant white light (70 µmol m^{-2} s^{-1}) at 21°C plus the indicated time in the stated conditions. Relative expression was assayed using RT-qPCR relative to the reference gene *IPP2* and normalized to the wild-type expression at 21WL 1h. The average values of three biological replicates per condition +/- SE are shown.
- **b.** Hypocotyl length of 3-day-old Arabidopsis seedlings of the indicated genotypes grown in constant blue light at 21°C (30 µmol m^{-2} s^{-1}). The number of seedlings (n) is shown under each box. Different letters denote statistical differences ($p < 0.05$) among samples as assessed by one-way ANOVA and Tukey HSD test. Boxes indicate the first and third quartiles and the whiskers indicate the minimum and maximum values, the black lines within the boxes indicate the median values and gray dots mark the individual measurements.

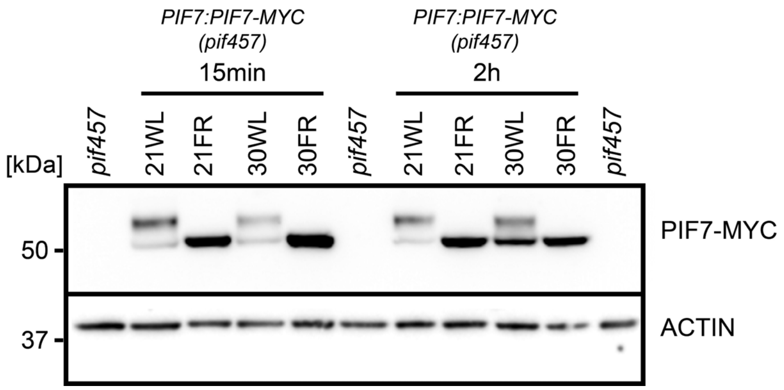

Supplementary Figure 5: PIF7 abundance at 21WL, 21FR, 30WL, or 30FR.

Representative immunodetection showing the abundance of PIF7-MYC Protein levels using anti-MYC antibody. Total protein extract collected from 3-day-old *PIF7:PIF7-4xMYC* (*pif457*) Arabidopsis seedlings grown in 21WL (70 μmol m^{-2} s^{-1}) plus 15 minutes or two hours in the stated conditions. Anti-ACTIN blot is shown as loading control. We repeated the experiment three times with similar results.

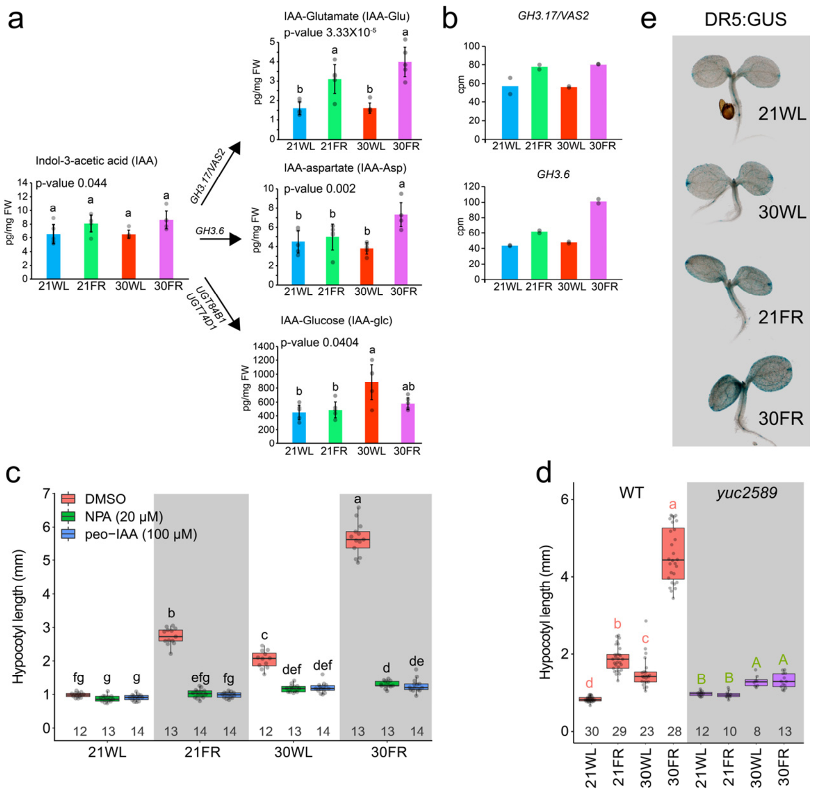

Supplementary Figure 6: Auxin transport and auxin signaling are crucial for hypocotyl elongation in response to low R/FR, warm temperature, and warm low R/FR conditions.

- **a.** Levels of free IAA, IAA-Glu, Iaa-Asp, and IAA-glc in Arabidopsis whole seedlings were grown in constant white light (70 µmol m^{-2} s^{-1}) at 21°C (21WL) for three days plus 3h in the indicated condition. Data show mean +/- SD. n = 5. The p-value of one-way ANOVA test are shown.
- **b.** Expression levels of *GH3.17/VAS2* and *GH3.6* derived from the RNA-seq after 3h of treatment. Data is shown as normalized counts per million mapped reads (cpm) and as the average of two biological replicates per condition. Gray dots mark the individual measurements.
- **c.** Hypocotyl length of 6-day-old wild-type Arabidopsis seedlings, grown on 20µM of NPA (polar auxin transport inhibitor) or 100µM peo-IAA (IAA antagonist). Seedlings were grown in constant white light (70 µmol m^{-2} s^{-1}) at 21°C for three days, then moved to plates with NPA or peo-IAA for an additional three days in the indicated condition. The number of seedlings (n) is shown under each box.
- **d.** Hypocotyl length of 6-day-old Arabidopsis seedlings of the indicated genotypes grown as described in Figure 1a. The number of seedlings (n) is shown under each box.
- **e.** Images of GUS-stained whole Arabidopsis seedlings carrying a DR5:GUS reporter. Plants were grown in constant LED white light (70 µmol m^{-2} s^{-1}) at 21°C plus 6h in the conditions indicated on the right.

In **a,c** and **d** different letters denote statistical differences ($p < 0.05$) within genotypes assessed by one-way ANOVA and Tukey HSD test. In **c** and **d** the boxes indicate the first and third quartiles and the whiskers indicate the minimum and maximum values, the black lines within the boxes indicate the median values and gray dots mark the individual measurements.

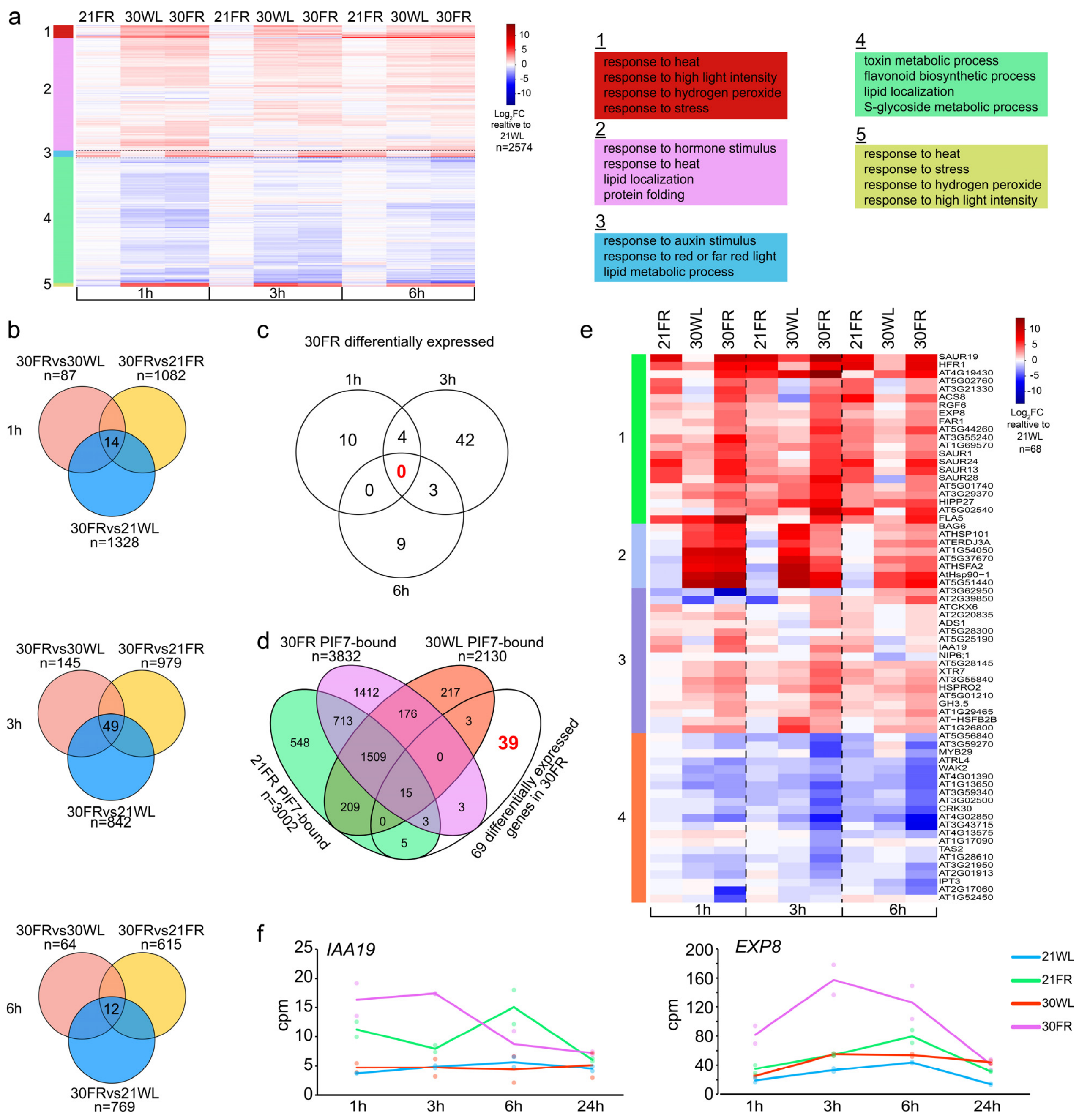

Supplementary Figure 7: Transcriptomic analysis reveals that the gene expression at 30FR resembles the combined gene expression profiles of 30WL and 21FR without a unique expression pattern.

- **a.** Expression profiles of differentially expressed genes (FDR<0.05 and FC > 1 or < -1) relative to 21WL in each time point are shown. Since most changes were seen within the first six hours, the 24h time point was removed from the cluster analysis. Some of the top enriched gene ontology processes in each cluster are shown on the left. For list of genes in each cluster see Supplementary Data 3.

b. Venn diagrams showing the number of genes differentially expressed in response to 30FR (FDR<0.05 and FC > 1 or < -1), relative to all other conditions (the list of genes in each time point can be found in Supplementary Data 3).

c. Venn diagram comparing the 30FR differentially expressed genes between all time points.

d. Venn diagram comparing the 30FR differentially expressed genes in the first six hours with the PIF7 bound genes in the different conditions. Bound genes were annotated if a PIF7 binding peak was found 2kb upstream of the transcriptional start site or in the gene body in both replicates. (n: number of genes found to be bound by PIF7, see Supplementary Data 4).

e. Expression profiles of the 30FR differentially expressed genes in the first six hours. For a list of genes in each cluster see Supplementary Data 3.

f. Expression of *IAA19* and *EXP*, shown as normalized counts per million mapped reads (cpm) and as the average of two biological replicates per condition. Dots mark the individual measurements.

a,**b**,**c,e,f** are based on the RNA-seq data from wild-type Arabidopsis seedlings grown as described in Figure 1a (see Methods section).

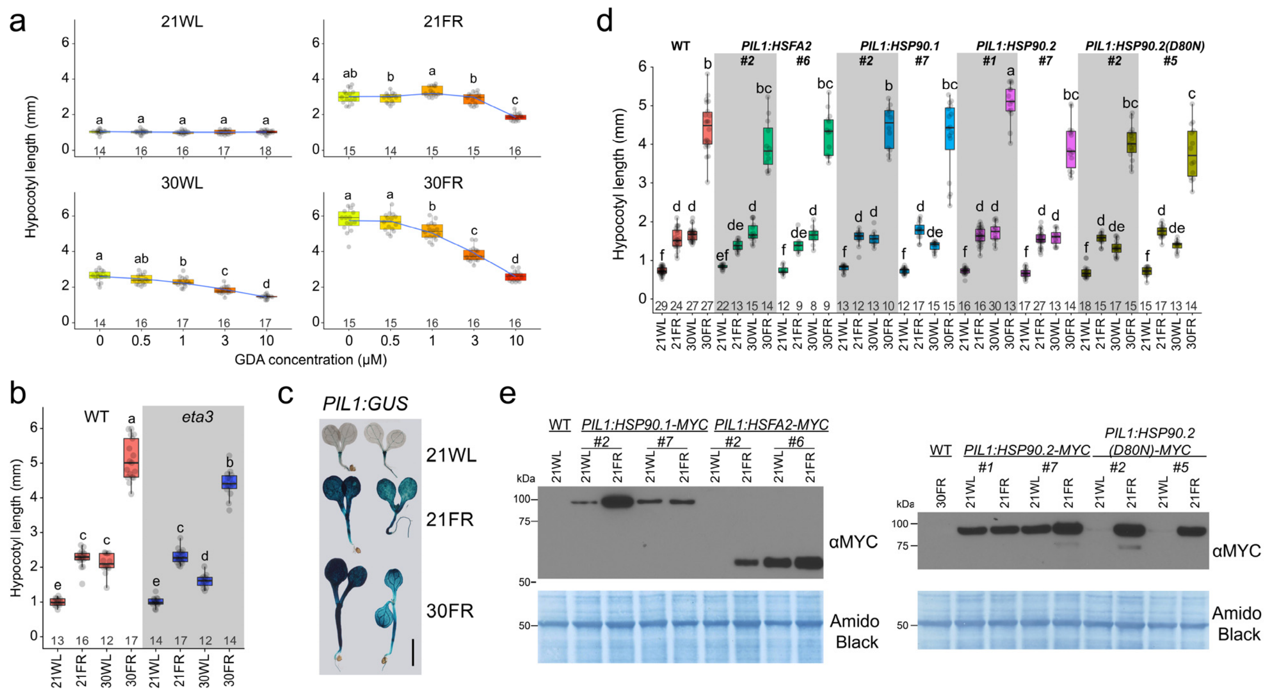

Supplementary Figure 8: The effect of HSP90 on the synergistic response to low R/FR and warm temperature.

- **a.** Dose-response curves of hypocotyl length for 6-day-old wild-type Arabidopsis seedlings, grown on Geldanamycin (GDA; HSP90 inhibitor). Seedlings were grown in constant WL (70 μmol m^{-2} s^{-1}) at 21°C for three days then moved to plates with GDA or DMSO control for an additional three days in the indicated condition. Data represent mean +/- SE, The number of seedlings (n) is shown under each box.
- **b.** Hypocotyl length of 6-day-old Arabidopsis wild-type and *eta3* mutant grown as described in Figure 1a. The number of seedlings (n) is shown under each box.
- **c.** Histochemical staining of β-glucuronidase (GUS) activity (blue). GUS was expressed under the control of the *PIL1* promoter. Shown are T1 Arabidopsis seedlings grown on hygromycin plates in constant WL (70 μmol m^{-2} s^{-1}) at 21°C for seven days, then moved to the indicated condition for three additional days. Bar = 5 mm.
- **d.** Hypocotyl length of 6-day-old Arabidopsis wild-type and *PIL1:HSFA2-MYC*, *PIL1:HSP90.1-MYC PIL1:HSP90.2-MYC* and the dominant-negative *PIL1:HSP90.2(D80N)-MYC* grown as described in Figure 1a. The number of seedlings (n) is shown under each box.
- **e.** Immuno-detection of PIL1:HSP90.1-MYC, PIL1:HSFA2-MYC (left blot), and PIL1:HSP90.2-MYC and the dominant-negative PIL1:HSP90.2(D80N)-MYC (right blot) using anti-MYC antibody. Total protein extract collected from 3-day-old seedlings grown in 21WL (70 μmol m^{-2} s^{-1}) plus 4 hours in indicated conditions. Amido black stained membranes are shown below as loading controls. This experiment was done one time.

In **a**, **b** and **d** different letters denote statistical differences ($p < 0.05$) within genotypes assessed by one-way ANOVA and Tukey HSD test. Boxes indicate the first and third quartiles and the whiskers indicate the minimum and maximum values, the black lines within the boxes indicate the median values and gray dots mark the individual measurements.

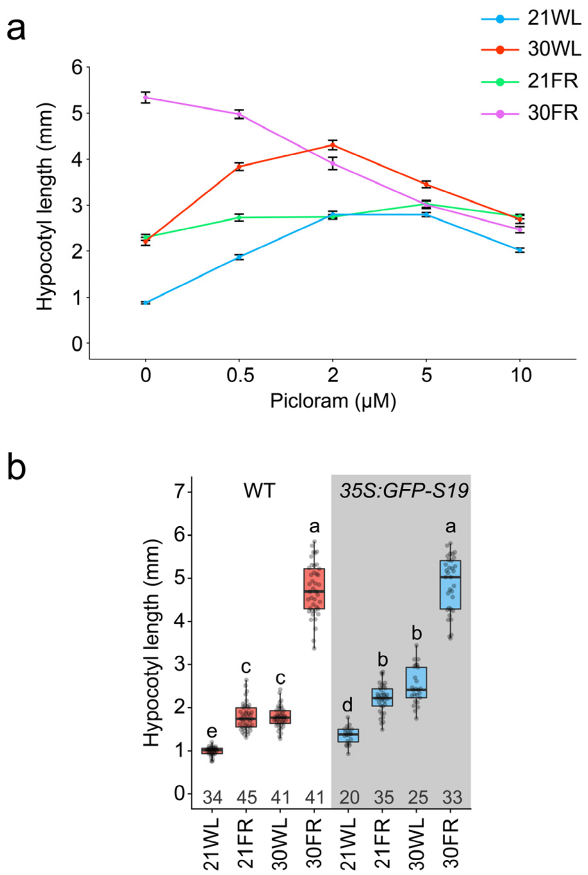

Supplementary Figure 9: The effect of picloram or *SAUR19* overexpression on the response to low R/FR and warm temperature.

- **a.** Dose-response curves showing hypocotyl length of 6-day-old wild-type Arabidopsis seedlings grown on the indicated concentrations of picloram, a synthetic auxin. Seedlings were grown in constant white light (70 µmol m^{-2} s^{-1}) at 21°C for three days then moved to plates with the indicated picloram concentration or DMSO control (0) for an additional three days. Data represent mean +/- SE; n = 15 seedlings or more per sample (for the exact number in each sample, see Source Data)..
- **b.** Hypocotyl length of 6-day-old Arabidopsis wild-type and *35S:GFP-SAUR19* grown as described in Figure 1a. Different letters denote statistical differences ($p < 0.05$) within genotypes assessed by one-way ANOVA and Tukey HSD. The number of seedlings (n) is shown under each box. Boxes indicate the first and third quartiles and the whiskers indicate the minimum and maximum values, the black lines within the boxes indicate the median values and gray dots mark the individual measurements.

Supplementary Table 1: Primers used in this work

Primer name	Sequence 5' >>> 3'	Purpose	Use
AttB4_PIL1p_F	GGGGACAACTTTGTATAGAAAAGTTGGAATTTAGAATGTGTCGAGAG	Cloning PIL1 promoter to pDONR P4-P1	Transformation into Arabidopsis
AttB1r_PIL1p_R	GGGGACTGCTTTTTTGTACAAACTTGCTGAAGTAAACTGAACAAAGCTTTCT		
AttB1_HSP90.1_F	GGGGACAAGTTTGTACAAAAAAGCAGGCTGCATGGCTGATGCAGAGACTTTTG	Cloning HSP90.1 to pDONR P1-P2	
AttB2_HSP90.1_R	GGGGACCACTTTGTACAAGAAAGCTGGGTGGTCGACTTCCTCCATCTTGCTC		
AttB1_HSP90.2_F	GGGGACAAGTTTGTACAAAAAAGCAGGCTCGATGGCGGACGCTGAAACCTTTG	Cloning HSP90.2 to pDONR P1-P2	
AttB2_HSP90.2_R	GGGGACCACTTTGTACAAGAAAGCTGGGTCGTCGACTTCCTCCATCTTGCTA		
AttB1-HSFA2_F	GGGGACAAGTTTGTACAAAAAAGCAGGCTGCATGGAAGAACTGAAAGTGGAAA	Cloning HSFA2 to pDONR P1-P2	
AttB2-HSFA2_R	GGGGACCACTTTGTACAAGAAAGCTGGGTGAGGTTCCGAACCAAGAAAAC		
D80N_F	ACCTTGACCATTATTAATAGTGGTATTGGCATG	Introduce point mutation to get HSP90.2(D80N)	
D80N_R	CATGCCAATACCACTATTAATAATGGTCAAGGT		
AttB2r-4XMYC	GGGGACAGCTTTCTTGTACAAAGTGGGCGATAACAGCGGGTTAATTAACGG	Cloning 4XMYC tag to pDONR P1-P2	
AttB3-4XMYC	GGGGACAACTTTGTATAATAAAGTTGCGGGGAAATTCGAGCTCTAAGC		
pif7-1_wt_mut	CATCCTCTGGTTTATCCTATCACGCCG	Genotyping *pif7-1*	Genotyping
pif7-1_wt	CCGTTCATGGTCTAGGCG		
pif7-1_mut	TGATAGTGACCTTAGGCGACTTTTGAACGC		
cry1-17_F	ACTTGCATTTTGGGGAAGTG	Sequnce *cry1-17* point mutation G→A Trp 400 Stop	
IPP2 real F	GTATGAGTTGCTTCTCCAGCAAAG	IPP2	qRT-PCR
IPP2 real R	GAGGATGGCTGCAACAAGTGT		
PIF7 real F	CGGAGCTTGAAGACAGCTAGAACC	PIF7	
PIF7 real R	GACTCGTTGTGAATCGCTGCTG		
PIF4 real F	CCAGATCATCTCCGACCGGTTTG	PIF4	
PIF4 real R	CTAGTGGTCCAAACGAGAACCGT		
PIF5 real F	GGGGTACAATCATCTCCATACAT	PIF5	
PIF5 real R	CCATGTACCTAGCGAGCTGCTCC		
PIL1 real F	TGGACTAATTCCAAACACTCCTATCTT	PIL1	
PIL1 real R	CACACGAAGGCACCACGA		
IAA29 real F	GGTGACAACTGCGAATACGTTACCA	IAA29	
IAA29 real R	CCCGGTAGCATCCGATCTTTTCA		
ATHB2 real F	GAGGTAGACTGCGAGTTCTTACG	ATHB2	
ATHB2 real R	GCATGTAGAACTGAGGAGAGAGC		
SAUR19 real F	CATACTTGAGCCAACCGTCA	SAUR19	
SAUR19 real R	GGATGAGCAAACCCAAACTC		
SAUR22 real F	TTTCATTCATCTTCAGATTTGCTT	SAUR22	
SAUR22 real R	CCAGAGCCATTTATAATTCTCTATTTG		
YUC8 real F	ATCAACCCTAAGTTCAACGAGTG	YUC8	
YUC8 real R	CTCCCGTAGCCACCACAAG		
YUC9 real F	TCTCTTGATCTTGCTAACCACAATGC	YUC9	
YUC9 real R	CCACTTCATCATCATCACTGAGATTCC		
