# Supplementary material for: PIF7 is a master regulator of thermomorphogenesis in shade
Source: Nat Commun. 2022 Aug 29;13:4942. doi: 10.1038/s41467-022-32585-6 (PMC9424238; doi:10.1038/s41467-022-32585-6)
Supplement: Supplementary file 3 — Description of Additional Supplementary Files [file 41467_2022_32585_MOESM3_ESM.pdf]

Description of Additional Supplementary Files

File Name: Supplementary Data 1
Description: Light conditions. Description of the light conditions which used in each of the experiments.

File Name: Supplementary Data 2
Description: RNA-seq data. Rna-seq data including normalized counts per million reads mapped (cpm) and Fold Change.

File Name: Supplementary Data 3
Description: Heat map clusters. List of genes present in the heat maps including GO analysis of each cluster.

File Name: Supplementary Data 4
Description: PIF7 peaks and annotated genes. PIF7-MYC ChIP-seq data including peak location and binding annotation.

File Name: Supplementary Data 5
Description: Measurements of free IAA and IAA-conjugates. LC-MS data set shows the calculation of the results.
