# Supplementary material for: PIF7 is a master regulator of thermomorphogenesis in shade
Source: Nat Commun. 2022 Aug 29;13:4942. doi: 10.1038/s41467-022-32585-6 (PMC9424238; doi:10.1038/s41467-022-32585-6)
Supplement: Supplementary file 9 — Reporting Summary [file 41467_2022_32585_MOESM9_ESM.pdf]

nature portfolio

Corresponding author(s): Yogev Burko

Last updated by author(s): Aug 4, 2022

Reporting Summary

Nature Portfolio wishes to improve the reproducibility of the work that we publish. This form provides structure for consistency and transparency in reporting. For further information on Nature Portfolio policies, see our Editorial Policies and the Editorial Policy Checklist.

Statistics

For all statistical analyses, confirm that the following items are present in the figure legend, table legend, main text, or Methods section.

n/a	Confirmed	
☐	☒	The exact sample size (*n*) for each experimental group/condition, given as a discrete number and unit of measurement
☒	☐	A statement on whether measurements were taken from distinct samples or whether the same sample was measured repeatedly
☐	☒	The statistical test(s) used AND whether they are one- or two-sided<br>*Only common tests should be described solely by name; describe more complex techniques in the Methods section.*
☒	☐	A description of all covariates tested
☒	☐	A description of any assumptions or corrections, such as tests of normality and adjustment for multiple comparisons
☐	☒	A full description of the statistical parameters including central tendency (e.g. means) or other basic estimates (e.g. regression coefficient) AND variation (e.g. standard deviation) or associated estimates of uncertainty (e.g. confidence intervals)
☐	☒	For null hypothesis testing, the test statistic (e.g. *F*, *t*, *r*) with confidence intervals, effect sizes, degrees of freedom and *P* value noted<br>*Give P values as exact values whenever suitable.*
☒	☐	For Bayesian analysis, information on the choice of priors and Markov chain Monte Carlo settings
☒	☐	For hierarchical and complex designs, identification of the appropriate level for tests and full reporting of outcomes
☒	☐	Estimates of effect sizes (e.g. Cohen's *d*, Pearson's *r*), indicating how they were calculated

Our web collection on statistics for biologists contains articles on many of the points above.

Software and code

Policy information about availability of computer code

Data collection	RNA-seq data was generated and collected on the Illumina HiSeq 2500 System
Data analysis	For RNA-seq: STAR (v2.5.4), HTSeq (v0.6.0), edgeR (v3.16.5)<br>For Hetamaps and plots: Heatmap.2, ggbeeswarm_0.6.0, ggplot2_3.3.5, reshape_0.8.8, cowplot_1.1.1, gplots_3.1.1, Rmisc_1.5<br>For GO analysis:agriGO v2.0<br>For measurements: ImageJ v1.50a (Fiji), Sapphire Biomolecular Imager and AzureSpot Pro (Azure Biosystems)<br>For T-test, Anova and significant difference in multiple comparisonTukeyHSD: stats, report_0.5.1, agricolae_1.3-5; For all R packages: R v4.1.0, RStudio v1.4.1717.<br>For qRT-PCR CFX384 Real-Time PCR Detection System and software were used.<br>To calculate free-IAA, IAA-Glutamate (IAA-Glu), IAA-Aspartate (IAA-Asp), and IAA-glucose (IAA-glc) levels the Agilent MassHunter Workstation quantitative analysis software (version B.05.02; Agilent Technologies) were used.

For manuscripts utilizing custom algorithms or software that are central to the research but not yet described in published literature, software must be made available to editors and reviewers. We strongly encourage code deposition in a community repository (e.g. GitHub). See the Nature Portfolio guidelines for submitting code & software for further information.

Data

Policy information about availability of data

All manuscripts must include a data availability statement. This statement should provide the following information, where applicable:

- Accession codes, unique identifiers, or web links for publicly available datasets
- A description of any restrictions on data availability
- For clinical datasets or third party data, please ensure that the statement adheres to our policy

RNA-seq (GSE196725) and ChIP-seq (GSE205210) data have been deposited into the Gene Expression Omnibus,
https://www.ncbi.nlm.nih.gov/geo/query/acc.cgi?acc=GSE196725
https://www.ncbi.nlm.nih.gov/geo/query/acc.cgi?acc=GSE205210

Field-specific reporting

Please select the one below that is the best fit for your research. If you are not sure, read the appropriate sections before making your selection.

☒ Life sciences ☐ Behavioural & social sciences ☐ Ecological, evolutionary & environmental sciences

For a reference copy of the document with all sections, see nature.com/documents/nr-reporting-summary-flat.pdf

Life sciences study design

All studies must disclose on these points even when the disclosure is negative.

Sample size	No statistical methods were used to calculate sample size. Sample sizes were defined based on previous work and were large enough for reproducible result. n values and statistical tests are presented in each figure, figure legend and source data.
Data exclusions	Seeds that field to germinate at the end of the experiment were excluded.
Replication	All of the experiments were repeated at least two times, and were reproduced successfully. A completely independent pool of side-by-side grown plants is considered as a biological replicate.
Randomization	we randomly arranged the plants with different genetic background in the growth chamber to account for potential position-based effects on plant growth.
Blinding	Not relevant to this study.

Reporting for specific materials, systems and methods

We require information from authors about some types of materials, experimental systems and methods used in many studies. Here, indicate whether each material, system or method listed is relevant to your study. If you are not sure if a list item applies to your research, read the appropriate section before selecting a response.

Materials & experimental systems

n/a	Involved in the study	
☐	☒	Antibodies
☒	☐	Eukaryotic cell lines
☒	☐	Palaeontology and archaeology
☒	☐	Animals and other organisms
☒	☐	Human research participants
☒	☐	Clinical data
☒	☐	Dual use research of concern

Methods

n/a	Involved in the study	
☐	☒	ChIP-seq
☒	☐	Flow cytometry
☒	☐	MRI-based neuroimaging

Antibodies

Antibodies used	anti-Myc, 9B11 (catalog no. 2276, Cell Signaling Technology)<br>anti-phyB monoclonal (gift from Akira Nagatani)<br>anti-Actin (catalog no. 2276, Sigma)<br>goat anti-mouse IgG (H + L)-HRP conjugate (catalog no. 1706516, Bio-Rad).
Validation	The data for Myc, Actin, and goat anti-mouse can be found on the manufacturer's website and for phyB in Shinomura et al., 1996.

Validation

phyB-9 mutant was used to validate the phyB antibodies and transgenic free plants were used to validate the MYC antibodies.

ChIP-seq

Data deposition

☒ Confirm that both raw and final processed data have been deposited in a public database such as GEO.

☒ Confirm that you have deposited or provided access to graph files (e.g. BED files) for the called peaks.

Data access links
May remain private before publication.

https://www.ncbi.nlm.nih.gov/geo/query/acc.cgi?acc=GSE205210

Files in database submission

Control_21FR.fastq
Control_30FR.fastq
PIF7_21WL_1.fastq
PIF7_21WL_2.fastq
PIF7_30WL_1.fastq
PIF7_30WL_2.fastq
PIF7_21FR_1.fastq
PIF7_21FR_2.fastq
PIF7_30FR_1.fastq
PIF7_30FR_2.fastq
Control_21FR_bedGraph.tdf
Control_30FR_bedGraph.tdf
PIF7_21WL_1_bedGraph.tdf
PIF7_21WL_2_bedGraph.tdf
PIF7_30WL_1_bedGraph.tdf
PIF7_30WL_2_bedGraph.tdf
PIF7_21FR_1_bedGraph.tdf
PIF7_21FR_2_bedGraph.tdf
PIF7_30FR_1_bedGraph.tdf
PIF7_30FR_2_bedGraph.tdf
Peaks and annotated genes.xlsx

Genome browser session
(e.g. UCSC)

no longer applicable

Methodology

Replicates

2 biological replicates from PIF7:PIF-4xMYC(pif457) and one from each control (Control_21FR and Control_30FR)

Sequencing depth

total reads uniquely mapped reads length
Control_21FR 40333649 5513540 single 101
Control_30FR 26402230 7324078 single 101
PIF7_21WL_1 38978600 10420024 single 101
PIF7_21WL_2 25795849 7586356 single 101
PIF7_30WL_1 40736539 12756414 single 101
PIF7_30WL_2 30476522 7541828 single 101
PIF7_21FR_1 64473089 24648532 single 101
PIF7_21FR_2 24583766 11612288 single 101
PIF7_30FR_1 37170785 7458745 single 101
PIF7_30FR_2 14933995 5814256 single 101

Antibodies

anti-Myc, 9B11 (Cat#2276, Cell Signaling Technology)

Peak calling parameters

Peaks were called using HOMER (version 4.11.1) Findpeaks script with the following setting: -style factor -F 2 -P 0.001 -L 2 -LP 0.001 for samples #1 (and control_21FR) -F 4 -P 0.0001 -L 4 -LP 0.001 for samples #2 (and control_30FR), IP from wild-type (Col-0) using anti-MYC antibody used as a background control (21FR or 30FR).

Data quality

Homer mergePeaks function used to find the peaks which presented in both biological replicate.

Software

Bowtie2 (version 2.2.5); HOMER (version 4.11.1); bedtools intersect (v2.30.0)
